# PD-L1 blockade engages tumor-infiltrating lymphocytes to co-express targetable activating and inhibitory receptors

**DOI:** 10.1186/s40425-019-0700-3

**Published:** 2019-08-14

**Authors:** Guillaume Beyrend, Esmé van der Gracht, Ayse Yilmaz, Suzanne van Duikeren, Marcel Camps, Thomas Höllt, Anna Vilanova, Vincent van Unen, Frits Koning, Noel F. C. C. de Miranda, Ramon Arens, Ferry Ossendorp

**Affiliations:** 1 Department of Immunohematology and Blood Transfusion, Leiden University Medical Center, Albinusdreef 2, 2333 ZA Leiden, The Netherlands; 2 Leiden Computational Biology Center, Leiden University Medical Center, Leiden, The Netherlands; 3 Department of Computer Graphics and Visualization Group, Delft, the Netherlands; 4 Computer Graphics and Visualization Group, faculty of Electrical Engineering, Mathematics and Computer Science, Delft University of Technology, Delft, the Netherlands; 5 Department of Pathology, Leiden University Medical Center, Leiden, The Netherlands

**Keywords:** Mass cytometry, Combinatorial immunotherapy, T cells

## Abstract

**Background:**

The clinical benefit of immunotherapeutic approaches against cancer has been well established although complete responses are only observed in a minority of patients. Combination immunotherapy offers an attractive avenue to develop more effective cancer therapies by improving the efficacy and duration of the tumor-specific T-cell response. Here, we aimed at deciphering the mechanisms governing the response to PD-1/PD-L1 checkpoint blockade to support the rational design of combination immunotherapy.

**Methods:**

Mice bearing subcutaneous MC-38 tumors were treated with blocking PD-L1 antibodies. To establish high-dimensional immune signatures of immunotherapy-specific responses, the tumor microenvironment was analyzed by CyTOF mass cytometry using 38 cellular markers. Findings were further examined and validated by flow cytometry and by functional in vivo experiments. Immune profiling was extended to the tumor microenvironment of colorectal cancer patients.

**Results:**

PD-L1 blockade induced selectively the expansion of tumor-infiltrating CD4^+^ and CD8^+^ T-cell subsets, co-expressing both activating (ICOS) and inhibitory (LAG-3, PD-1) molecules. By therapeutically co-targeting these molecules on the T_AI_ cell subsets in vivo by agonistic and antagonist antibodies, we were able to enhance PD-L1 blockade therapy as evidenced by an increased number of T_AI_ cells within the tumor micro-environment and improved tumor protection. Moreover, T_AI_ cells were also found in the tumor-microenvironment of colorectal cancer patients.

**Conclusions:**

This study shows the presence of T cell subsets in the tumor micro-environment expressing both activating and inhibitory receptors. These T_AI_ cells can be targeted by combined immunotherapy leading to improved survival.

**Electronic supplementary material:**

The online version of this article (10.1186/s40425-019-0700-3) contains supplementary material, which is available to authorized users.

## Introduction

Immunotherapy has become an important treatment option for cancer patients. Especially, clinical trials with antibodies that block the interaction between the inhibitory receptor PD-1, expressed on previously activated T cells, with its broadly expressed ligand PD-L1, resulted in unprecedented clinical response rates for patients with advanced cancer [1–3]. Despite these encouraging results, still only a fraction of patients show durable responses, whereas the majority of treated patients show no beneficial clinical response [1, 4]. Therefore, there is a need for more effective treatment regimens, like combinatorial immunotherapies, which offer an attractive avenue to improve the efficacy and the duration of the tumor specific T-cell response.

Both CD8^+^ and CD4^+^ T cells can mount responses against many human cancer types, especially those with higher mutational burden [5]. Studies have shown that T cells are however partially inhibited by PD-1/PD-L1 interactions [6] and releasing this constraint by blocking the PD-1 pathway can to some extent reinvigorate T cells leading to clinical benefit in a number of cancer patients [7]. However, tumor-specific T cells are also restrained by several other inhibitory mechanisms [8, 9], which put forward the premise that PD-1/PD-L1-based monotherapies could be enhanced so that the majority of patients will have durable clinical benefit. Indeed, recent studies reported co-treatment regimens to PD-1 blockade [10–13]. In depth mechanistic studies of PD-1/PD-L1 blockade in vivo may lead to rational design of improved co-treatment protocols.

Identification of biomarkers related to immunotherapeutic response and resistance could support the rational design of complementary therapies in which the additional targeting of those biomarkers would lead to more effective cancer therapies. Identification of the relevant responding cell types to therapy reveals insight into the underlying immunological mechanisms of on-going clinical response, as well as into the development of adaptive resistance during such a therapy. Here we used high-dimensional, single-cell mass cytometry and a customized bioinformatics pipeline *Cytofast* [14] to generate an in-depth analysis of the tumor-infiltrating immune cells upon PD-L1-based treatment. Our aim was to identify responsiveness-associated targets to improve immunotherapy. We discovered unique CD4^+^ and CD8^+^ T cell subsets that increased after anti-PD-L1 immunotherapy and were characterized by expression of both **a**ctivating and **i**nhibitory receptors, hence we defined these cells as T_AI_ cells. By therapeutic targeting of the activating and inhibitory receptors on the T_AI_ cells in vivo, significant improvement of immunotherapy was shown, correlating with an increase of the CD8^+^ T_AI_ cells in the tumor micro-environment (TME). T_AI_ cells were also present within tumor-infiltrated immune cells from mismatch repair-deficient (MMRd) colorectal cancer patients. Together, our data show the importance of the T_AI_ cells and their possible targetability to induce tumor regression in colorectal cancer.

## Methods

### Mice

C57BL/6 J mice were purchased from The Jackson Laboratory. All animal experiments were approved by the Animal Experiments Committee of LUMC and were executed according to the animal experimentation guidelines of the LUMC in compliance with the guidelines of Dutch and European committees.

### Staining and acquisition for CyTOF mass cytometry

Metal conjugated antibodies were purchased from Fluidigm or conjugated to unlabeled antibodies in-house. All non-platinum conjugations were performed using X8 polymer as per manufacturer’s protocol (Fluidigm) and were performed at 100 μg scale. Conjugation with 208 Bismuth was performed using a protocol adapted from M. Spitzer [15]. All in-house conjugated antibodies were diluted to 0.5 mg/ml in antibody stabilizer supplemented with 0.05% sodium azide (Candor Biosciences). Appropriate antibody dilution was determined by serial dilution staining to minimize background and optimize detection of positively expressing populations.

CyTOF data were acquired and analyzed on-the-fly, using dual-count mode and noise-reduction on. All other settings were either default settings or optimized with tuning solution, as instructed by Fluidigm Sciences. After data acquisition, the mass bead signal was used to normalize the short-term signal fluctuations with the reference EQ passport P13H2302 during the course of each experiment and the bead events were removed [16].

### CyTOF mass cytometry data analysis

To isolate immune cells from the tumor, solid tumors were excised after a flushing step to remove the blood from TME. Exclusion criteria were ulceration of tumors, incomplete or unsuccessful flushing (determined by an unexpected high numbers of B cells in the TME). Single-cell suspensions were then prepared by mechanical and enzymatic (collagenase D and DNase, Sigma-Aldrich) dissociation, followed by density gradient centrifugation on an 100% / 70% / 40% / 30% Percoll (GE Healthcare) gradient.

After staining cells according to van Unen et al. [17], we analyzed live immune cells from the TME. We set our gating strategy to live single cells, positive for CD45, and excluded reference beads. For further analysis, live CD45^+^ gated files were sample-tagged, their marker expression arcsinh5 transformed and subjected to dimensionality reduction analyzes in Cytosplore [18]. All markers were taken in account to process the clustering analysis except PD-L1, which is a marker used only as a quality control to check the efficacy of PD-L1 blocking antibodies. The PD-L1 blocking antibody we used (clone MIH5, rat-anti-mouse, IgG2a subtype) binds to FcyRIIb and FcyRIII but not to FcyRI and FcyRIV, and is not able to mediate specific killing or depletion [19]. By staining with the same antibody clone, PD-L1 downmodulation was determined to show the effectiveness of the provided therapeutic antibodies to block PD-L1 binding.

Pooled samples from control and PD-L1 treated groups were analyzed by hierarchical stochastic neighbourhood embedding (HSNE) [20] based on approximated t-distributed stochastic neighbourhood embedding (A-tSNE) [21]. The default perplexity and iterations of the HSNE analysis were 30 and 1.000, respectively. If some clusters showed a similar phenotype, they were manually merged in Cytosplore. For further data exploration, CD4^+^ T cell, CD8^+^ T cell, CD19^+^ B cell, CD11b^+^ myeloid cell lineages were analyzed separately. Downstream analysis was performed by *Cytofast* [14] and *Cytofworkflow* [22].

### Diffusion map

Diffusion map was generated with R using the *cytofkit* package [23] by displaying only CD3^+^ metaclusters identified by PhenoGraph [24] as a confirmation method of the HSNE clustering.

### Reference standard comparison

Reference standard samples were compared with each other by calculating the similarity between their respective t-SNE maps. We used the Jensen-Shannon (JS) divergence to quantify the similarity between t-SNE maps. After converting t-SNE maps into two-dimensional probability density functions, the similarity between two maps is quantified as the JS divergence between their corresponding probability density functions. We used the base 2 logarithm in the JS divergence computation, which results in a continuous range of JS divergence values between 0 (for identical distributions) and 1 (for fully disjoint distributions), the algorithm being provided by E.D. Amir [25]. The average overlap frequency (AOF) is determined as described by E.D. Amir [26]

### Flow cytometry

#### Mouse

Single-cell suspensions were prepared from TME [27] obtained from untreated or PD-L1 treated mice by an incubation of 15 min with collagenase and DNase IV (Roche) and by mincing the tumor tissue through a 70-μm cell strainer (BD Bioscience). Live cells were washed with RPMI-1640 supplemented with 8% FBS and P/S and once with FACS buffer. Subsequently, samples were incubated with Fc block mouse (2%) and mouse serum (5%) for 10 min, then stained with antibodies (Additional file 1: Table S1A**)** for 30 min at 4 °C in the dark and finally rinsed two times with PBS containing 0.5% BSA solution. Samples were acquired using the LSR Fortessa (BD Biosciences) and results analyzed with FlowJo and Cytosplore software.

#### Granzyme B staining of tumor-infiltrated T cells

MC-38 tumors were injected subcutaneously in C57BL/6 J mice, consecutively treated with 200 μg PD-L1 at three different timepoints (10, 13 and 16 days after tumor inoculation). At day 8 post treatment, tumors were excised and single-cell suspensions were generated as described above. Cells were next stimulated overnight in vitro with MC-38 tumor cells with a concentration of Brefeldine A of 4 μg/mL. Cells were then cell surface stained with antibodies to CD45, CD3, CD8, CD4, PD-1 and CD39 followed by intracellular Granzyme B staining after fixation. The phenotype was assessed by flow cytometry using the LSR Fortessa and results were analyzed with FlowJo.

#### Human studies

Cryopreserved colorectal tumor digests (excision and single cell suspension preparation by mechanical dissociation followed by slow freezing in 10% DMSO) were thawed and mashed through 70 μm filters into RPMI-1640 supplemented with 8% FBS and P/S. Live cells were washed once with RPMI-1640 with 8% FBS and P/S and once with FACS buffer. Two staining reactions of 1 × 10^6^ cells per tumor sample were analyzed. All samples were then incubated with 2% of each bovine, murine, rat, hamster, and rabbit serum PBS with human TruStain FcX (Biolegend, 422,302) at 4 °C for 10 min. Samples were processed for surface staining (Additional file 1: Table S1B) and analyzed using a similar protocol as described for processing, staining and analyzing murine tumor samples. All specimens were anonymized and handled according to the ethical guidelines described in the Code for Proper Secondary Use of Human Tissue in the Netherlands of the Dutch Federation of Medical Scientific Societies.

### In vivo murine tumor experiments

MC-38 colon adenocarcinoma cells were injected at a dose of 0.3 × 10^6^ cells subcutaneously (s.c.) in the right flank. Antibodies blocking LAG-3 and PD-L1 were injected intraperitoneally and agonistic anti-ICOS antibodies were given subcutaneously, next to the tumor. Tumor diameter was measured every 2 to 3 days with a calliper and reported as volume using the formula (w × h × l) x (π/6).

### Statistical analyses

Statistical analyses were performed using R software or Prism (GraphPad). Unpaired two-tailed t-tests were used for subset abundance comparisons.

## Results

### Efficacy of PD-L1 blockade parallels with an increase in tumor infiltrating CD8^+^ T cells over time

**Fig. 1:**
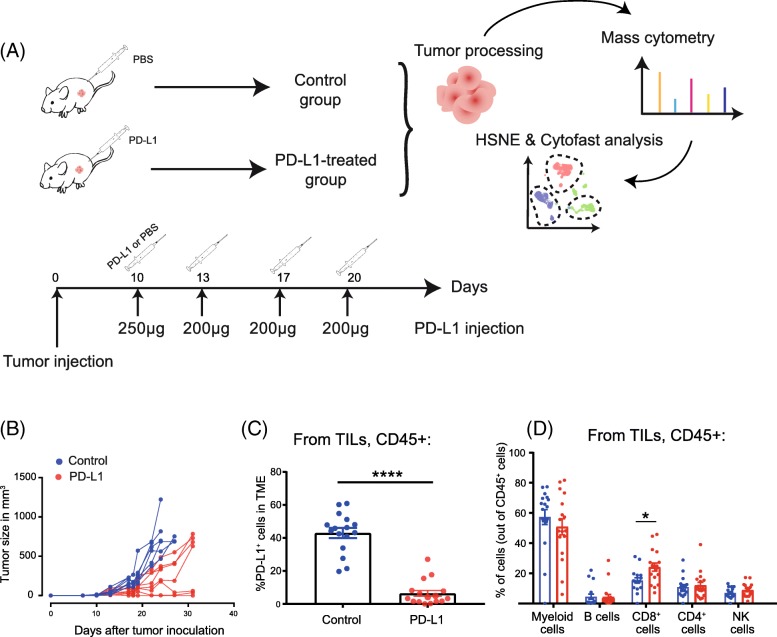
PD-L1-blocking treatment induces delay of MC-38 tumor growth. (**a**) Schematic of CyTOF mass cytometry experiment investigating the effect of PD-L1 antibody treatment on the TME. Mice were challenged with MC-38 tumor cells and subsequently tumor-bearing mice were treated either with PBS (*n* = 16 mice) or PD-L1 blocking antibodies (n = 16 mice). Tumors were isolated and analyzed by mass cytometry (CyTOF). Cluster identification was performed with HSNE and downstream analysis was done with *Cytofast*. (**b**) Tumor growth curves of individual mice in the control group (mock injected with PBS, blue lines) and PD-L1-treated group (red lines). (**c**) Frequency of CD45^+^ PD-L1^+^ cells in the TME 8 days after therapy starts displayed on a per-mouse basis with mean ± SEM. (**d**) Overview of the immune cell composition in the TME shown in percentage of cells on a per-mouse basis with mean ± SEM (n = 16 mice per group)

To examine the effect of PD-L1 blocking therapy, we used the colorectal adenocarcinoma mouse model MC-38. Mice were inoculated with MC-38 tumor cells, and when tumors were established after 10 days (tumor volume of 30–40 mm^3^), mice were treated with PD-L1 blockade therapy or left untreated (control group) (Fig. 1A**)**. To identify biomarkers that respond to immunotherapy with PD-L1 blockade, we set up a CyTOF mass cytometry panel for in-depth phenotypic characterization of tumor-infiltrated lymphocytes (TILs) in preclinical tumor models, which allows kinetic dissection of anti-tumor immune responses. The panel consisted of 38 cell surface markers and was designed to identify the major lymphoid and myeloid subsets and to ascertain the differentiation and activation status of these subsets (Additional file 1: Figure S1). We isolated immune cells from the tumor 8 days after start of immunotherapy and stained the single cell suspensions followed by mass cytometry acquisition of 3.5 million cells in total. In parallel, tumor growth was measured to assess the therapeutic benefit of the PD-L1 blockade treatment. Treated animals displayed a significant delay in tumor progression or even had complete tumor eradication (Fig. 1B). To determine the effectiveness of the provided therapeutic antibodies to block PD-L1 binding, the cell surface expression of PD-L1 in the TME was assessed by staining with the same antibody clone (i.e. MIH5). Indeed, the PD-L1 expression on CD45^+^ tumor-infiltrated immune cells from the treated group was significantly decreased compared to control animals (Fig. 1C).

To monitor the robustness of the measurement, we included reference standard acquisitions and used the Jensen-Shannon (JS) divergence calculation to determine similarity between samples. The results yielded consistency between the measurements with low JS distance, meaning high similarities between samples (Additional file 1: Figure S2A). We also tested the quality of our staining by using the Average Overlap Frequency (AOF), a metric to evaluate and quantify the robustness of staining and clustering quality in high-dimensional data [26]. Importantly, all the markers involved in the cluster identification of CD3^+^ cells (e.g. CD4, CD8, PD-1, ICOS, etc.) showed an AOF < 0.3, which indicates a valid staining of the samples and a clear separation between negative and positive signals (Additional file 1: Figure S2B). Together, these data showed a stable and reliable sample acquisition with limited inter-sample variation.

An overview of the main tumor-infiltrating immune cells identified by mass cytometry showed a higher proportion of CD8^+^ T cells in the PD-L1 treated group (24.1%) compared to the control group (16.1%) 8 days after first injection **(**Fig. 1D**)**. Simultaneously, the frequency of the CD11b^+^ myeloid compartment decreased after PD-L1 blockade. Thus, PD-L1 blockade empowers the increase of CD8^+^ T cells and limits the infiltration of myeloid cells in the TME.

### PD-L1 treatment increases selectively CD8^+^ T-cell subsets expressing both activating and inhibitory receptors

**Fig. 2:**
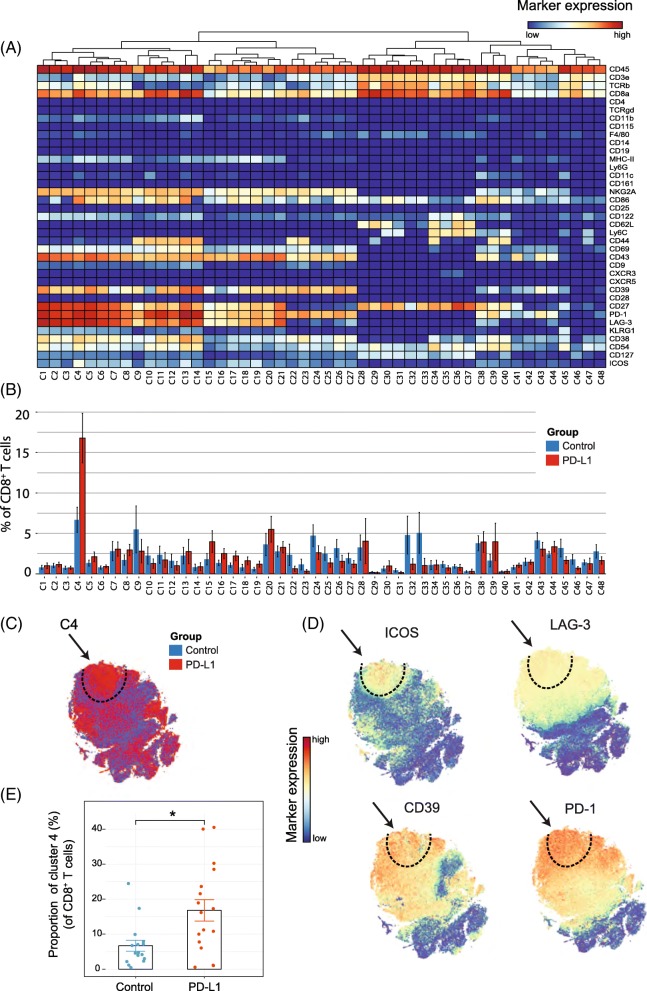
Identification of CD8^+^ T-cell clusters in Tumor Infiltrating T-cell populations (**a**) Heatmap of all CD8^+^ T-cell clusters identified at day 8 after the start of the PD-L1 treatment. Data shown is based on t-SNE plots, and is pooled from the control and PD-L1 treated group. Level of ArcSinh5-transformed expression marker is displayed by a rainbow scale. Dendrogram on the top represents the hierarchical similarity between the identified clusters. (**b**) Average and SEM in percentage of each CD8^+^ T-cell cluster among the CD8^+^ T-cell population of control (blue bars) and PD-L1 group (red bars). (**c**) t-SNE plot of respectively 0.32 × 10^6^ and 0.35 × 10^6^ CD8^+^ T cells from control (blue) and PD-L1 treated (red) group. (**d**) Same t-SNE plots as above now showing the level of expression marker by a rainbow scale. The arrow identifies the cluster of interest C4 (having a shared CD8^+^ LAG3^+^ ICOS^+^ phenotype). (**e**) Bar graph showing the mean frequency of cluster 4 (± SEM, unpaired t-test). Individual mice belonging to the control (blue) and PD-L1 treated (red) group are indicated

Because treatment with anti-PD-L1 has major effects on the expansion of the CD8^+^ T-cell compartment, we analyzed in detail the CD8^+^ TIL subset at this timepoint and identified 48 different CD8^+^ T-cell subsets (Fig. 2A). t-SNE clustering allowed distinction between naïve (e.g. cluster C28 expressing CD62L, CD27), effector (e.g. cluster C13 and C14 expressing CD54, CD38, CD27, CD44) and central-memory subsets (e.g. cluster C34 expressing CD54, CD62L, CD44, CD27). Remarkably, one cluster (cluster C4) displayed both activating (ICOS, CD69, CD43) and inhibitory receptors (PD-1, LAG-3, NKG2A). To visualize the distribution of each identified cluster, we displayed the abundance of each subset per treatment group (Fig. 2B). The t-SNE map overlaid with the expression of specific markers showed that the cluster C4 subset could be defined by the inhibitory molecule LAG-3 and the costimulatory receptor ICOS. Essentially, co-expression of ICOS and LAG-3 was highly specific to the PD-L1 blockade treated group (Fig. 2C, D). Further characterization of this subset also demonstrated up-regulation of the ectonucleotidase CD39, the early activation marker CD69, the inhibitory NKG2A receptor, and the activation/exhaustion cell surface marker PD-1. The CD8^+^ T-cell subset expressing both the **a**ctivating and **i**nhibitory molecules, referred hereafter as T_AI_ cells, represented approximately 17% of all the CD8^+^ T cells across individual mice in the PD-L1 blockade group compared to 7% in the control group (Fig. 2E). Next, we validated the presence of CD8^+^ T_AI_ cells by flow cytometry. We isolated TILs from the TME and stained for the markers ICOS, LAG-3, CD69, CD39 and PD-1. The CD8^+^ T_AI_ subset (CD8^+^, LAG-3^+^, CD39^+^, PD-1^+^, ICOS^+^) population could indeed be identified, and was more abundant following PD-L1 blockade therapy (mean = 22%, sd = 16%, *n* = 6) than in the untreated group (mean = 9%, sd = 8%, n = 6; *p*-value = 0.03 by Student’s t-test). In addition, we confirmed our findings in the MCA205 sarcoma model. We identified the CD8^+^ T_AI_ cells by flow cytometry and observed that PD-L1 treatment increased this subset as compared to the control untreated group **(**Additional file 1: Figure S3A**)**.

### Identification of T_AI_ cell subsets in the tumor-infiltrated CD4^+^ T-cell compartment

**Fig. 3:**
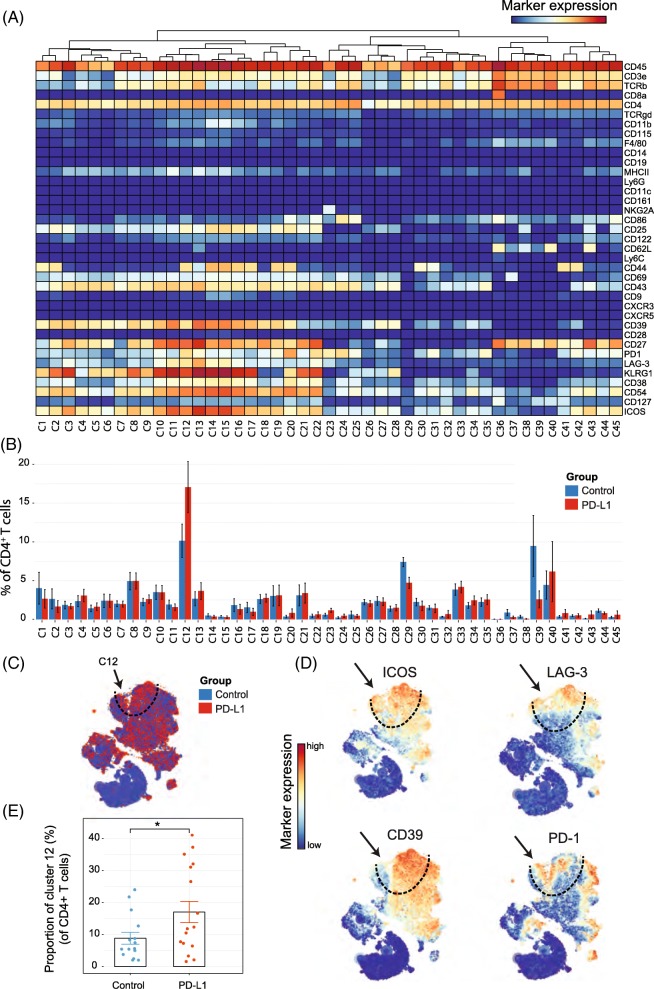
Identification of CD4^+^ T-cell clusters in Tumor Infiltrating T-cell populations (**a**) Heatmap of all CD4^+^ T-cell clusters identified at day 8 after the start of the PD-L1 treatment. Data shown is based on t-SNE plots, and is pooled from the control and PD-L1 treated group. Level of ArcSinh5-transformed expression marker is displayed by a rainbow scale. Dendrogram on the top represents the hierarchical similarity between the identified clusters. (**b**) Average and SEM in percentage of each CD4^+^ T-cell cluster among the CD4^+^ T-cell population of control (blue bars) and PD-L1 group (red bars). (**c**) t-SNE plot of respectively 0.23 × 10^6^ and 0.25 × 10^6^ CD4^+^ T cells from control (blue) and PD-L1 treated (red) group. (**d**) Same t-SNE plots as above now showing the level of expression marker by a rainbow scale. The arrow identifies the cluster of interest 12 (having a shared CD4^+^ LAG3^+^ ICOS^+^ phenotype) (**e**) Bar graph showing the mean frequency of cluster 12 (± SEM, unpaired t-test). Individual mice belonging to the control (blue) and PD-L1 treated (red) group are indicated.

We next analyzed whether PD-L1 blockade therapy-specific subsets were also apparent in the CD4^+^ T-cell compartment. The t-SNE algorithm identified 45 CD4^+^ T-cell subsets revealing the heterogeneous profile of the CD4^+^ T cells (Fig. 3A, B). Notably, as for the CD8^+^ T cells, one subset was identified that correlated with PD-L1 treatment (cluster C12) and displayed the activating molecule ICOS and the inhibitory molecule LAG-3. In addition, these CD4^+^ T_AI_ cells expressed CD27, CD39, CD43, CD44, CD54, KLRG1 and PD-1. The t-SNE map overlaid with the expression of specific markers showed that these subsets could also be defined by LAG-3, ICOS and CD39 and the co-expression of those markers was highly specific to the PD-L1 treated group (Fig. 3C, D). The T_AI_ subset of CD4^+^ T cells was also significantly more abundant, representing about 17% of the total CD4^+^ T-cell population within the tumor infiltrated immune cells of the treated group compared to 8% in the control group (Fig. 3E). Also, in the MCA205 tumor model, the CD4^+^ T_AI_ cells were identified and were increased by PD-L1 treatment **(**Additional file 1: Figure S3B).

### Differentiation relationships of the identified PD-L1 treatment-associated T-cell subsets

**Fig. 4:**
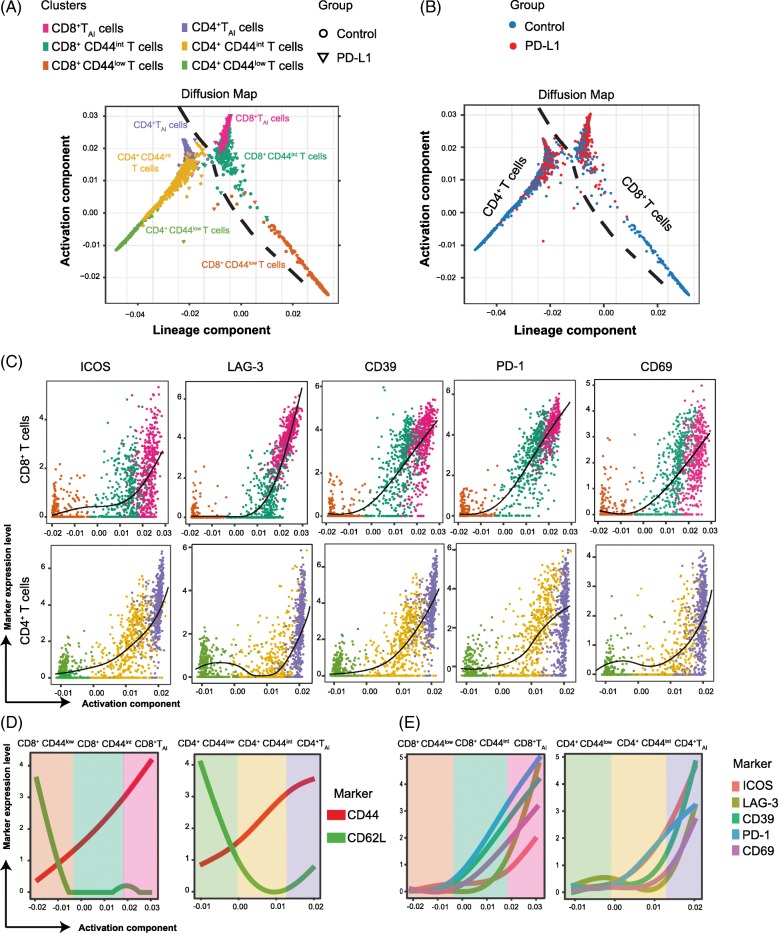
Diffusion maps of the identified CD4^+^ and CD8^+^ subsets in the control and treated group. (**a**) Two-dimensional diffusion map of the CD4^+^ and CD8^+^ T cells present in the tumor at day 8 after the first PD-L1 treatment. Three different CD4^+^ and CD8^+^ T cell metaclusters have been identified by PhenoGraph. Continuity of the pattern reveals relationship between the different represented metaclusters (*n* = 5 mice per group). (**b**) Diffusion map of the CD4^+^ and CD8^+^ T_AI_ cell displayed by group origin (PBS in blue and PD-L1 in red). (**c**) Diffusion map of the CD4^+^ and CD8^+^ T_AI_ cell displayed by marker expression ICOS, LAG-3, CD39, PD-1 and CD69. (**d**) Expression levels of CD44 and CD62L on the metaclustered CD4^+^ and CD8^+^T cell populations. (**e**) Expression levels of ICOS, LAG-3, CD39, PD-1 and CD69 on the metaclustered CD4^+^ and CD8^+^T cell populations

To corroborate the results obtained from the previous t-SNE analysis regarding the PD-L1 treatment-associated T-cell subsets, we used the PhenoGraph algorithm to identify cell clusters and their differentiation status [24]. Similar T-cell metaclusters as those depicted by t-SNE earlier were indeed identified **(**Fig. 4A**)**. The CD4 and CD8 T-cell lineages could be distinguished into a resting phenotype (called *CD44*^*low*^*)*, an activated intermediate phenotype without inhibitory marker expression (called *CD44*^*int*^), and the T_AI_ cells expressing both inhibitory and activation molecules (called *T*_*AI*_). To investigate the relationship between those metaclusters identified by PhenoGraph, we used the diffusion map algorithm [28].

The two represented components defined gradual trends of variation (Fig. 4A) correlated with signatures for lineage and activation. Both CD4^+^ and CD8^+^ T cells could be distinguished on the diffusion map, showing the independent differentiation lineages of CD4^+^ and CD8^+^ T cells. The T_AI_ cells (CD39^+^, PD-1^+^, LAG-3^+^, ICOS^+^), more frequent in the PD-L1 treated group (Fig. 4B), could be derived from an intermediate phenotype, which was CD44^int^. Thus, due to PD-L1 blockade treatment, T cells further differentiate into the more activated T_AI_ phenotype.

We next analyzed the level of expression of the individual activating and inhibitory molecules that were modulated upon anti-PD-L1 therapy. By displaying the diffusion map with the expression level (Fig. 4C), we observed that the expression of ICOS, LAG-3 and CD39 started to be upregulated on intermediate phenotypes but maximum expression of these molecules was reached on both CD4^+^ and CD8^+^ T_AI_ cells.

A summary of the phenotype of the three different clusters studied is represented by the evolution of the markers CD62L and CD44 (Fig. 4D). While PD-1 expression was more prominent on CD8^+^ T_AI_ cells, ICOS was more abundantly expressed on CD4^+^ T_AI_ cells (Fig. 4E). The inhibitory and activating markers NKG2A, CD38 and CD43 were also found to be upregulated on the CD8^+^ T_AI_ cell subset (data not shown).

### Early induction of CD4^+^ T_AI_ and CD8^+^ T_AI_ cells upon PD-L1 blocking

**Fig. 5:**
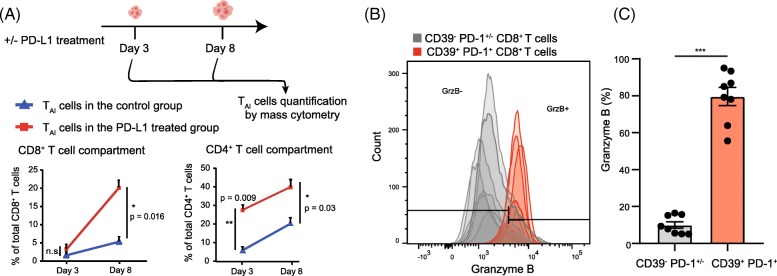
Quantification and cytotoxic capacity of the T_AI_ cells in the TME (**a**) Average percentage (and SEM) of T_AI_ cells within the CD8^+^ (left panel) and CD4^+^ (right panel) T-cell compartment at day 3 and 8 post PD-L1 blockade therapy in MC-38 tumor challenged mice. (**b**) Granzyme B expression of CD8^+^ T cell subsets at day 8 post PD-L1 treatment in MC-38 tumor bearing mice. The grey shaded histograms represent the CD39^−^ PD-1^+/−^ CD8^+^ T cells and the red shaded histograms depict the CD39^+^ PD-1^+^ CD8^+^ T_AI_ cells of individual mice. (**c**) The percentage of granzyme B^+^ cells among the CD39^+^ PD-1^+^ CD8^+^ T_AI_ cells after 8 days of PD-L1 treatment in the MC-38 tumor model compared to the CD39^−^ PD-1^+/−^ CD8 T cells

PD-L1 blocking treatment enhanced CD4^+^ and CD8^+^ T_AI_ cell subsets in the TME 8 days post therapy. To determine if the expansion of these compartments occurred already early after treatment, we analyzed the TME at day 3 post-treatment (i.e. 13 days after tumor inoculation). The expansion of the CD4^+^ T_AI_ cells started at an earlier stage, 3 days post-therapy, and continued over time. The presence of the CD8^+^ T_AI_ cells could also be observed 3 days after the start of the treatment, but these cells significantly increased over time (Fig. 5A). Essentially, the vast majority of the CD39^+^ PD1^+^ CD8^+^ T cells that are present in the TME produce copious amounts of granzyme B, revealing their cytotoxic potential (Fig. 5B).

### Rational design of combinatorial immunotherapy targeting activating and inhibitory receptors

**Fig. 6:**
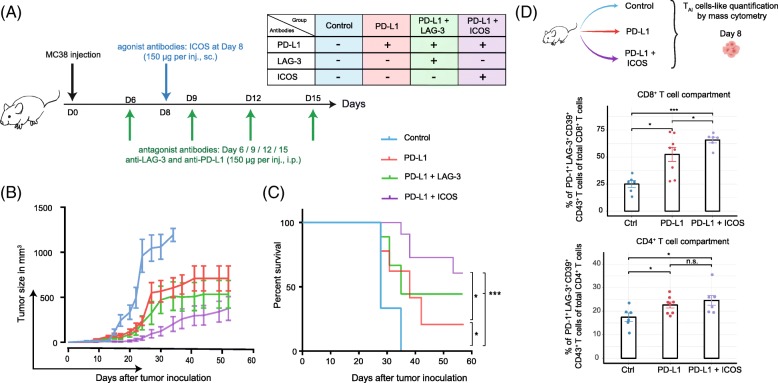
Correlation between the presence of T_AI_ cells in the TME and tumor growth. (**a**) Regimen scheme of (combinatorial) antibody treatment after tumor injection. (**b**) Comparison of tumor growth between control group (*n* = 9), PD-L1 antibody treated group (n = 9), PD-L1 and ICOS antibody treated group (*n* = 11), PD-L1 and LAG-3 antibody treated group (*n* = 10). (**c**) Survival curves for each treatment mentioned above. (**d**) Study of the tumor-microenvironment after control (*n* = 6), single therapy (PD-L1, *n* = 8) or combinatorial therapy (PD-L1 and ICOS, n = 6) of the CD8^+^ T_AI_-like cells (left panel) and CD4^+^ T_AI_-like cells (right panel) at day 8 (unpaired t-test) displayed on a per-mouse basis with mean ± SEM

The data above indicate that the activity of anti-PD-L1 treatment could be mediated via the expansion of CD4^+^ and CD8^+^ T_AI_ cells that express activating receptors and inhibitory receptors. We assessed if we could further enhance the functionality of the T_AI_ cells by combining the PD-L1 blockade treatment with antibodies targeting inhibitory and stimulatory molecules. For the proof-of-principle, we performed co-treatment studies with blocking antibodies to the inhibitory receptor LAG-3 and with agonistic antibodies to ICOS during PD-L1 blockade (Fig. 6A).

PD-L1 blockade therapy in combination with LAG-3 blockade resulted in enhanced survival and tumor growth delay. Co-treatment with agonistic ICOS antibody improved PD-L1 blockade therapy even further (Fig. 6B-C**,** Additional file 1:Figure S4).

Next, we aimed to examine whether induction of T_AI_ cells is linked to the improved survival rate observed in the PD-L1 plus ICOS targeting combination therapy. At day 8 after single and combined therapy, we analyzed the TME, and specifically analyzed the T_AI_ cell abundance in each tumor. Because, in vivo treatment with ICOS antibodies prevents ex vivo staining for ICOS, we defined the T_AI_ cells with the markers PD-1, CD39 and CD43. The percentage of CD8^+^ T_AI_ cells was significantly higher in the PD-L1 blockade treated group compared to the control group. Importantly, significantly higher percentages of CD8^+^ T_AI_ cells were observed in mice treated by the combined ICOS and PD-L1 targeted therapy compared to control or PD-L1 blockade treated mice. Expansion of CD4^+^ T_AI_ cells upon single and combinatorial therapy was equivalent (Fig. 6D). Thus, combinatorial therapy targeting ICOS and PD-L1 expands CD8^+^ T_AI_ cells and relates to improved survival of the treated mice.

### Identification of T_AI_ cells in human colorectal cancer

**Fig. 7:**
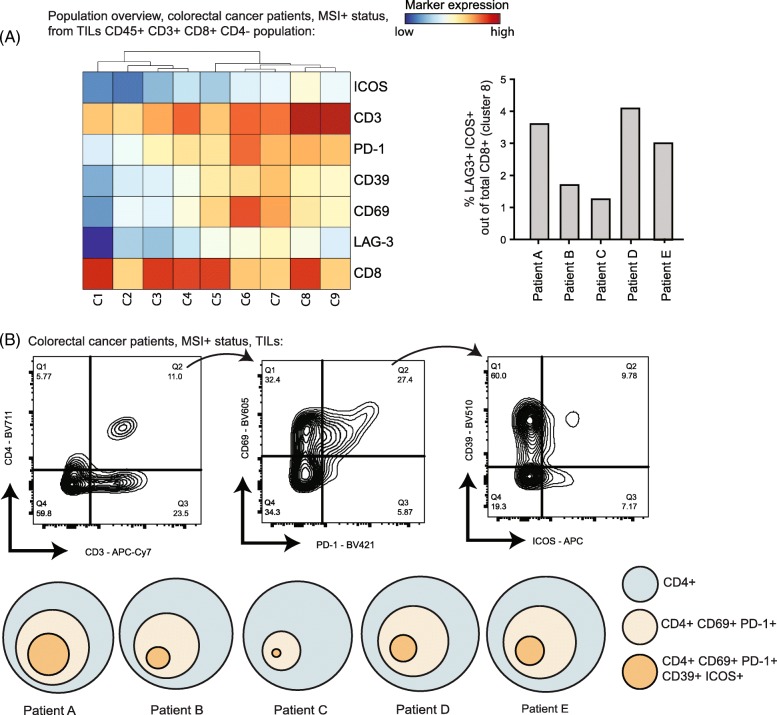
Identification of the T_AI_ cell subset in humans. (**a**) Heatmap of CD8^+^ T-cell phenotypes (pre-gated on CD45^+^ CD3^+^ CD4^−^ cells) in tumors of 5 human colorectal cancer (MMRd) patients. Dendrogram above shows the hierarchical similarity between the identified clusters. Right panel shows the frequency of CD8^+^ LAG3^+^ ICOS^+^ cells (cluster 8) among total CD8^+^ T cells across the 5 patients. (**b**) Gating strategy to identify the CD4^+^ CD69^+^ PD1^+^ CD39^+^ ICOS^+^ population in human colorectal cancer. The abundance proportional to circle area is shown

To extrapolate our findings in preclinical models to clinical settings, we questioned whether the T_AI_ cells were present within tumor-infiltrated immune cell populations in human tumors. We investigated the phenotype of the TILs in colorectal tumors of five patients, who have not undergone any immunotherapy. To reflect the immunogenicity of the MC-38 model, we selected MMRd colorectal cancer patients [29]. We designed our flow cytometry panel to characterize putative T_AI_ subsets within the tumor infiltrated CD8^+^ and CD4^+^ T cells. Hence, we included the activating receptors ICOS and CD69, also the inhibitory receptors like LAG-3 and CD39. We depicted the CD8^+^ T cells phenotypic diversity by gating on CD45^+^ CD8^+^ CD4^−^ cells and showed that a subset (cluster 8) with a similar phenotype (CD69^+^ ICOS^+^ and LAG-3^+^) as identified in mice tumors could be found in human tumors (Fig. 7A). The CD4^+^ T cell pool in human tumors contained a substantial fraction of cells with a CD69^+^ PD1^+^ phenotype, and within this population a CD39^+^ ICOS^+^ subset could be identified (Fig. 7B). Together, these results established that in tumors of mice and humans, CD4^+^ and CD8^+^ T_AI_ cell subsets are present.

## Discussion

Variance of clinical outcomes upon checkpoint blocking immunotherapy like PD-L1 antibody treatment reflects the diversity of the anti-tumor immune response. In the current work, we identified the expansion of CD4^+^ and CD8^+^ T-cell subsets that strikingly co-expressed both inhibitory markers, like PD-1 and LAG-3, and activating markers like ICOS. These subsets, named T_AI_ cells, expanded over time, starting 3 days after therapy and were still visible 8 days after the start of the therapy. Since the PD-L1 blocking antibody we used does not induce antibody-dependent cell-mediated cytoxicity [19], the expansion of the T_AI_ cells is most likely caused by blocking the PD-1 signaling pathways rather than e.g. depleting PD-L1^+^ cells or reactions to the antibody itself.

The T_AI_ cells appear to play a central role in mediating tumor rejection, despite the expression of inhibitory receptors. The variance seen in response to PD-L1 therapy could be explained by a variable expansion of T_AI_ cells in the TME and needs to be further explored. Our unbiased high dimensional immunophenotyping of the TME provides a deeper insight on the immune changes triggered by immune checkpoint blockade. By identifying a precise expansion of specific subsets in the TME, this strategy enabled us to rationally design immunotherapeutic combination treatments. We were able to enhance the anti-tumor efficacy of PD-L1 blocking therapy by combining it with an agonist ICOS therapy or an antagonist LAG-3 therapy. The T_AI_ cells identified in our murine models shared a similar phenotype with colorectal cancer patients and therefore a similar effect of the combination therapy could be expected. Hence, this detection of the T_AI_ cells in human tumors could thus pave the path to clinically target these cells in colorectal cancer by e.g. combined PD-1/PD-L1 and ICOS targeted immunotherapy. We surmise that TIL analysis by mass cytometry might be a powerful tool for personal-guided combinatorial therapy for each individual patient.

Our mass cytometry panel only screened for certain immunomodulatory molecules of the CD28 superfamily. Upregulation of other molecules, as has been reported for CTLA-4 [30] or BTLA, might have occurred but were not analyzed due to the limitation of the number of markers in our designed mass cytometry panel. On the other hand, we have included other markers like LAG-3, CD39, CD38, NKG2A, CD43, CD54, ICOS, KLRG1, which have never been analyzed at once in mass cytometry on ex vivo TILs. A large percentage of the T_AI_ cells may be tumor-reactive and have encountered tumor-specific antigenic peptides (e.g. neo-antigens). The granzyme B expression within the T_AI_ cells underlines this and is consistent with previous work showing that CD39 expression is a marker for cancer-related CD8^+^ T cells in the TME [31]. Consistently, CD8^+^ T cells expressing PD-1 have also been shown to be more reactive against tumors [32].

Our study is in line with previous studies on other tumor models like the T3 methylcholanthrene-induced sarcomas showing that inhibitory markers like PD-1 and TIM-3 and activating receptors like ICOS are co-expressed on tumor-specific T cells [33]. In addition, it was found that the expansion of CD8^+^ T cells expressing PD-1 improves the efficacy of adoptive T-cell therapy [34] and T cells co-expressing CD39 and PD-1 or LAG-3 and PD-1 were found to expand after anti-PD-1 therapy [7, 35].

Remarkably, in a viral setting, CD8^+^ T cells that provide the proliferative burst after PD-1 therapy are expressing ICOS [36], suggesting that the T_AI_ cell expansion in the TME relies on the co-expression of ICOS and PD-1 markers. PD-1 and ICOS are also co-expressed on T cells in human bladder tumors [37]. Our results can also explain the positive correlation between higher ICOS expression and a better overall survival in colorectal cancer patients [38]. Together, this is strengthening the relevance of targeting the PD-1^+^ ICOS^+^ T_AI_ cells by the above-mentioned dual therapy targeting PD-L1 and ICOS. Interestingly, ICOS appears to be relatively higher expressed on CD4^+^ T_AI_ cells than on CD8^+^ T_AI_ cells, which we aim to further explore. The T_AI_ cells expanding after PD-L1 blocking therapy also co-expressed LAG-3, which might explain the better efficiency of the combination of targeting PD-L1 and LAG-3. These findings are coherent with what has been previously reported in other studies [39, 40].

The T_AI_ cells are intratumorally present at an early stage, irrespective of the treatment and respond to immunotherapy as shown by an increase in the TME across time. This suggests that the T_AI_ cells are an identifiable unique subset among T cells, existing before immunotherapy, which can be further expanded by treatment. Tracking these cells in the TME warrants further investigation and would inform about their origin and the plasticity of their phenotype.

The expansion kinetics of the CD4^+^ T_AI_ cells compared to the CD8^+^ T_AI_ cells after PD-L1 treatment are dissimilar. In both relative abundance and absolute numbers CD4^+^ T_AI_ cells are already strongly expanded at day 3 after treatment in contrast to CD8^+^ T_AI_ cells, while at day 8 the CD8^+^ T_AI_ cells are more expanded. This is in line with a restored early helper function of the CD4 compartment to stimulate expansion of effector CD8^+^ T cells. Immunotherapy in the MC-38 model is fully dependent on CD8^+^ T cells [41]. Indeed, after 8 days of PD-L1 treatment, regression of tumor size becomes apparent. We could confirm that similar tumor infiltrating T-cell subsets exist in colorectal cancer patients. CD4^+^ T_AI_ subsets co-expressing inhibitory PD-1 and activating ICOS as well as CD39 and CD69 were detectable in freshly resected colon tumor from MMRd colorectal cancer patients known to express neo-epitopes due to accumulated point-mutations. It would be interesting to study these T_AI_ subpopulations in patients upon treatment with checkpoint therapy or other immunotherapies.

The relevance of targeting simultaneously inhibitory and activating molecules is already transposed in humans. For example, three clinical trials ongoing (NCT02904226, NCT02723955 and NCT02520791) are meant to study the effect of anti-ICOS as monotherapy or in combination with anti-PD-1. Our preclinical study suggests the synergistic effect of ICOS together with a blocking PD-L1 therapy. A systematic immunophenotyping of the TME should enable a better prediction of response to immunotherapy and a progress in development of rational immunotherapeutic strategies.

## Conclusion

This study described the expansion of a treatment-related cell subset, named T_AI_ cells, which co-express activating and inhibitory molecules. In preclinical mouse models, both CD4^+^ and CD8^+^ T_AI_ cells were higher in abundance in the TME upon PD-L1 therapy. Co-targeting the inhibitory receptor LAG-3 or the activating receptor ICOS on the T_AI_ cells further enhanced this subset and resulted in improved tumor immunity. T_AI_ cells were also present in human colorectal tumors. We surmise that targeting the inhibitory and activating receptors on these T_AI_ cells could lead to enhanced tumor immunity.

## Additional file

Additional file 1 **: Figure S1**. Mass cytometry panel and marker expression. **Figure S2.** Quality control assessment of the data generated by mass cytometry. **Figure S3**. Identification of CD4^+^ and CD8^+^ T_AI_ cells in the MCA205 sarcoma model. **Figure S4**. Synergy of combination immunotherapy. **Table S1**. FACS panels used in the study. (DOCX 2099 kb)

## Data Availability

All data generated or analyzed during this study are included in this published article.

## Abbreviations

AOF: Average Overlap Frequency
A-tSNE: Approximated t-distributed Stochastic Neighbor Embedding
HSNE: Hierarchical Stochastic Neighbor Embedding
MMRd: MisMatch Repair-deficient
TILs: Tumor Infiltrated Lymphocytes
TME: Tumor Micro-Environment
t-SNE: t-distributed Stochastic Neighbor Embedding
