# Supplementary material for: PD-L1 blockade engages tumor-infiltrating lymphocytes to co-express targetable activating and inhibitory receptors
Source: J Immunother Cancer. 2019 Aug 14;7:217. doi: 10.1186/s40425-019-0700-3 (PMC6694641; doi:10.1186/s40425-019-0700-3)
Supplement: Supplementary file 1 — : Figure S1. Mass cytometry panel and marker expression. Figure S2. Quality control assessment of the data generated by mass cytometry. Figure S3. Identification of CD4+ and CD8+ TAI cells in the MCA205 sarcoma model. Figure S4. Synergy of combination immunotherapy. Table S1. FACS panels used in the study. (DOCX 2099 kb) [file 40425_2019_700_MOESM1_ESM.docx]

**SUPPLEMENTARY FIGURES – Beyrend et al.**

**
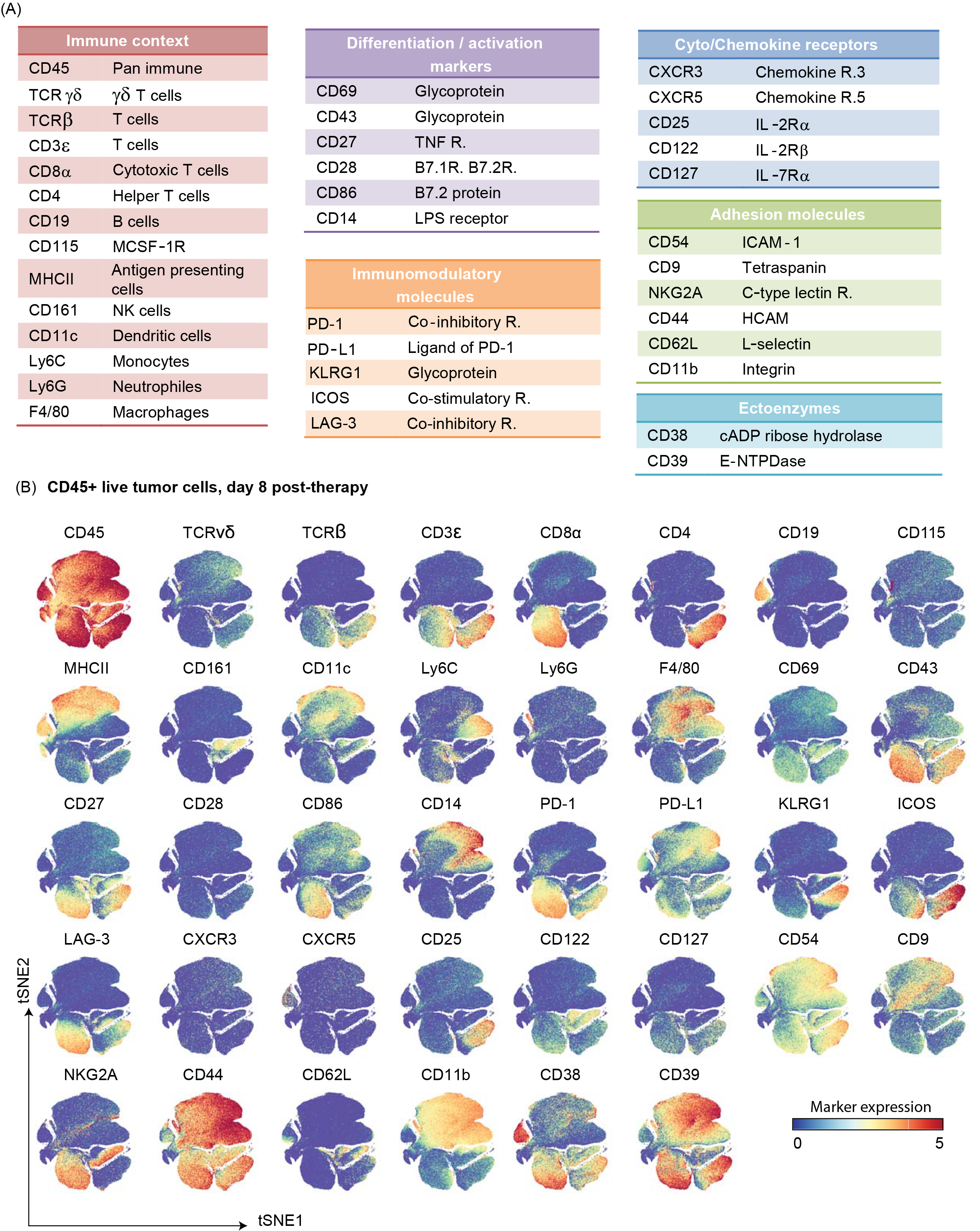
**

**Figure S1. Mass cytometry panel and marker expression**

(A) Description of the mass cytometry panel.

(B) t-SNE-based two-dimensional maps showing ArcSinh5-transformed marker expression patterns (rainbow scale) of each antibody on live CD45^+^ cells (4.0×10^4^) present in MC-38 tumors, 8 days post PD-L1therapy. Each dot represents a single cell.


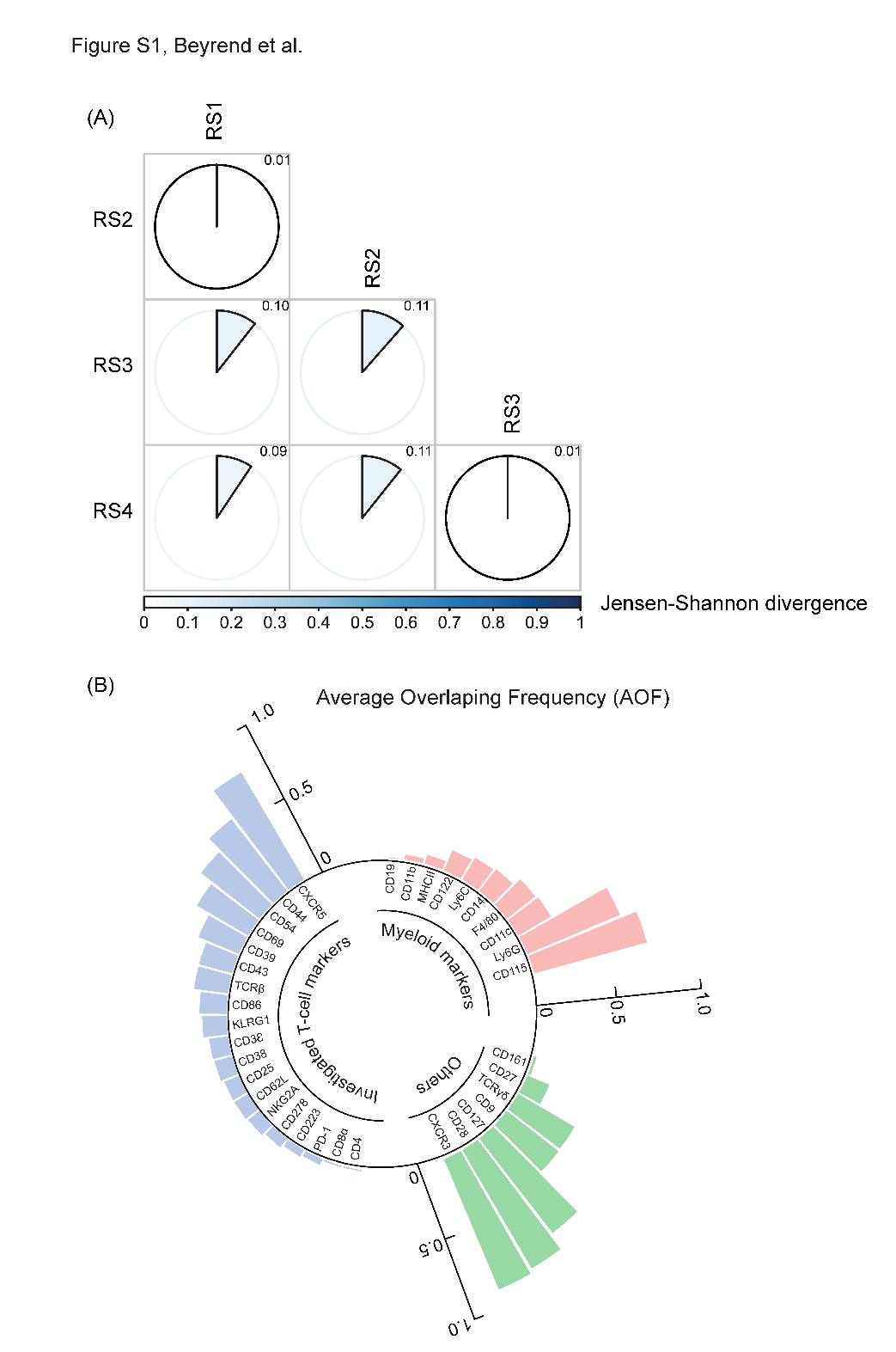


**Figure S2. Quality control assessment of the data generated by mass cytometry**

(A) Matrix displaying the Jensen-Shannon divergence of the reference standard measured at regular intervals. All values are low, showing consistency between all measurements.

(B) Bar plots representing the Average Overlap Frequency values for CD3^+^ events in the TME (myeloid markers shown in pink, T-cell markers in blue and others in green). In bold are highlighted the markers discussed in Figure 3. Values close to zero indicates a clear separation between the positive and negative peaks.


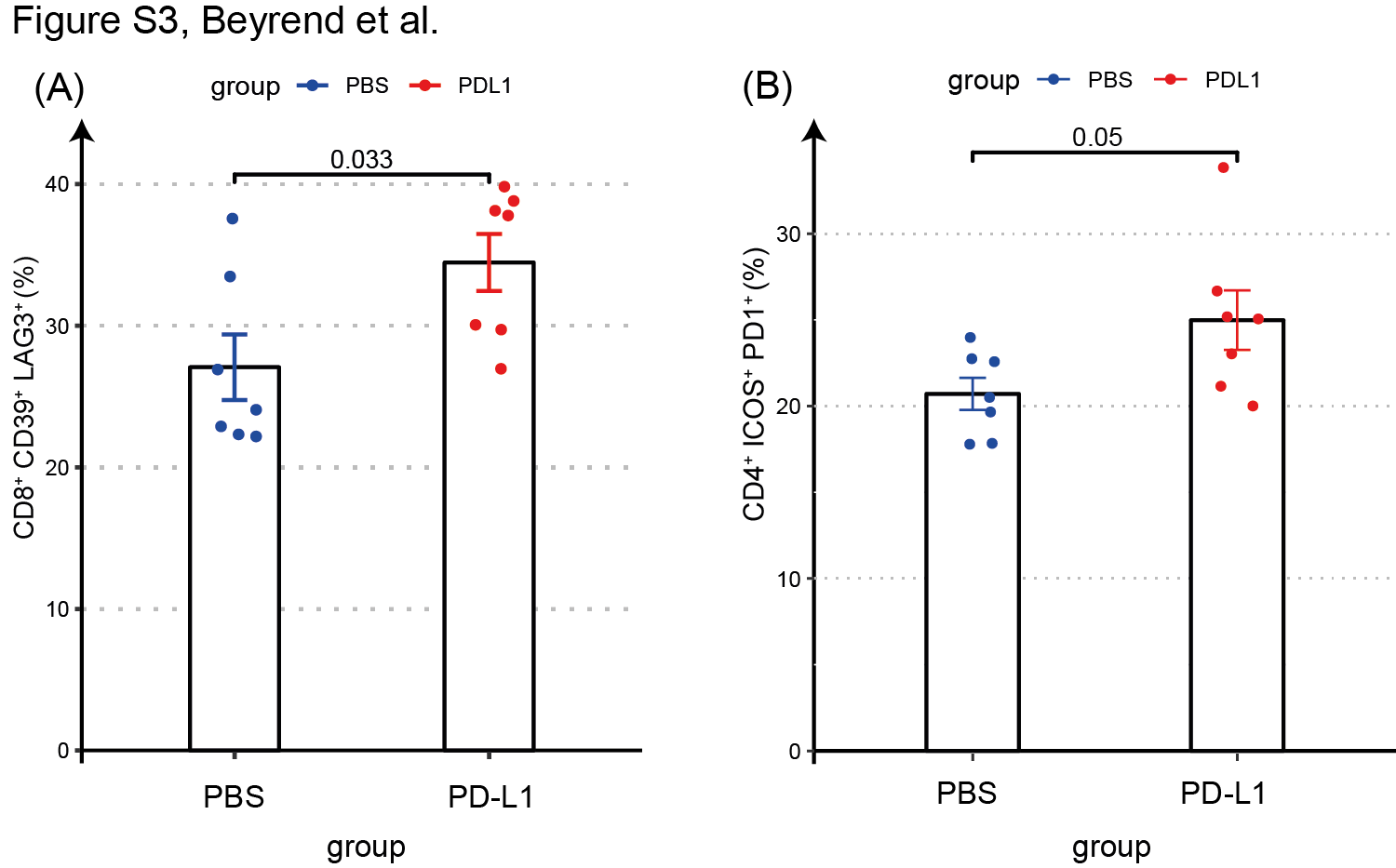


Figure S3. Cytotoxic capacity of CD8^+^ T_AI_ cells and identification of CD4^+^ and CD8^+^ T_AI_ cells in the MCA205 sarcoma model.

(A) Bar graph showing the percentage of CD8^+^ T_AI_ cells in the MCA205 tumor model. CD8^+^ T_AI_ cells were identified by CD39 and LAG-3 expression at day 8 post treatment (control (blue) and PD-L1 treated group (red)).

(B) Bar graph showing the percentage of CD4^+^ T_AI_ cells in the MCA205 tumor model. CD4^+^ T_AI_ cells were identified by ICOS and PD-1 expression at day 8 post treatment (control (blue) and PD-L1 treated group (red)).


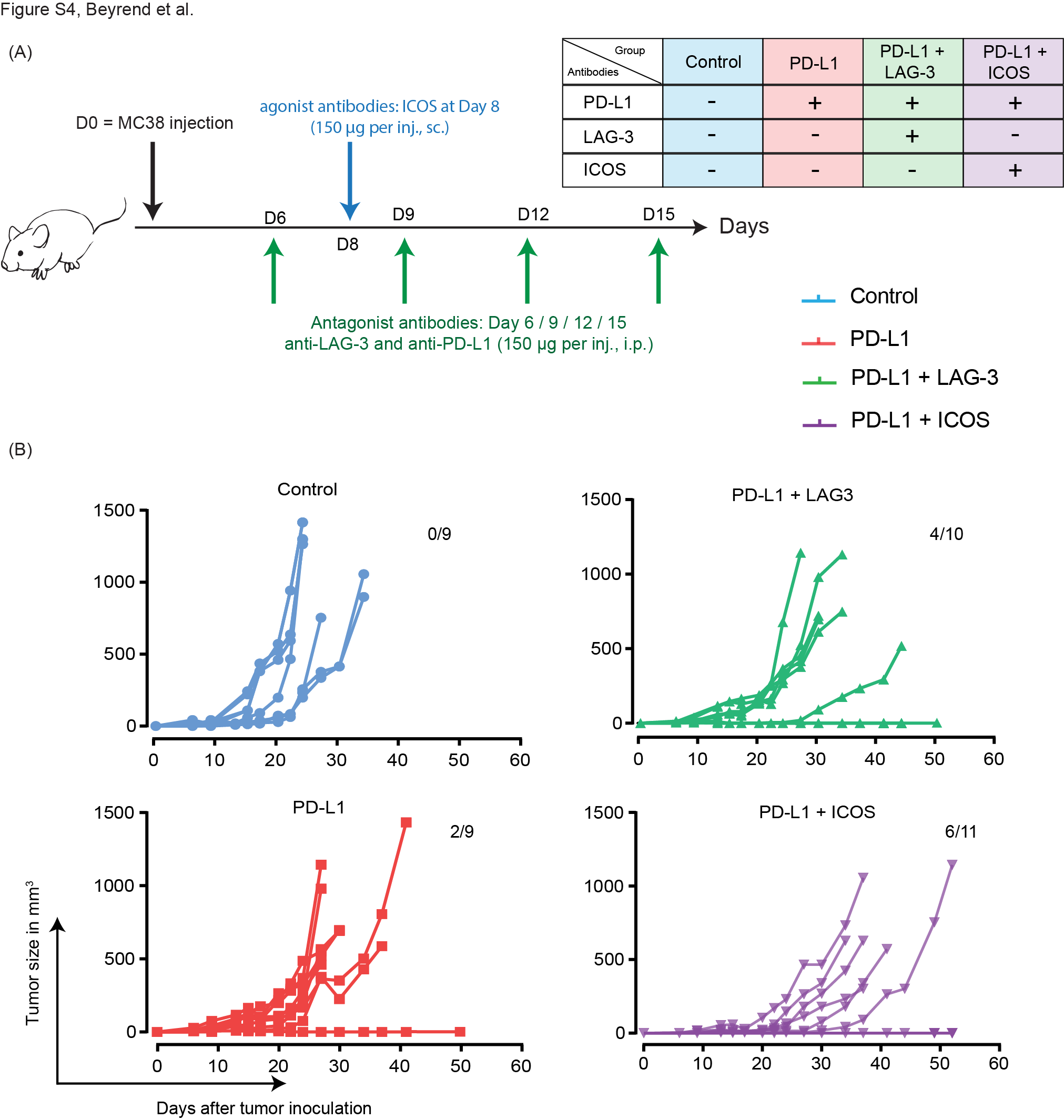


**Figure S4. Synergy of combination immunotherapy.**

(A) Schematic of (combination) treatment after tumor challenge.

(B) Individual tumor growth curves of control (PBS, n=9), PD-L1 treated (n=9), PD-L1 and LAG-3 treated (n=10), and PD-L1 and ICOS treated mice (n=11). At day 32, mice from the combination groups PD-L1 + LAG-3 and PD-L1 + ICOS presented smaller tumors compared to the control group (respectively p=0.02 and p <0.0001, unpaired t-test) or compared to the single therapy PD-L1 (respectively p=0.08 and p<00.6, unpaired t-test).

**Table S1. FACS panels used in the study.**

| **Table S1A.** | | |  |  |
| --- | --- | --- | --- | --- |
| **FACS panel for mouse studies** | | |  |  |
| **Antibodies** | **Clone** | **Color** | **Manufacturer** | **Reference** |
| CD4 | RM4-5 | BV605 | BD Biosciences | 563151 |
| CD8 | 53-6.7 | Alexa 700 | BioLegend | 100730 |
| CD45 | 104 | FITC | BD Biosciences | 553772 |
| 7AAD | 7AAD | PerCP5.5 | ThermoFisher | A1310 |
| CD3 | 500A2 | BV500 | BD Biosciences | 550277 |
| ICOS | 7E.17G9 | PE | BioLegend | 117406 |
| LAG3 | C9B7W | PE-Cy7 | BioLegend | 125226 |
| CD25 | PC61 | APC | BioLegend | 102012 |
|  |  |  |  |  |
| **Table S1B.** | | |  |  |
| **FACS panel for human studies** | | |  |  |
| **Antibodies** | **Clone** | **Color** | **Manufacturer** | **Reference** |
| CD45 | 30-F11 | FITC | BD Biosciences | 345808 |
| LAG3 | FAB2319P | PE | R&D | FAB100A |
| ICOS | ISA-3 | APC | ThermoFisher | 17-9948-41 |
| CD8 | RPA-T8 | PE-Cy7 | BD Biosciences | 557746 |
| CD3 | 17A2 | APC eFluor | ThermoFisher | 47-0038-41 |
| PD1 | EH12.2H7 | BV421 | Biolegend | 329919 |
| CD39 | A1 | BV510 | Sony Biotechnology | 2241095 |
| 7AAD | 7AAD | Per-CP5.5 | ThermoFisher | A1310 |
| CD4 | SK3 | BV711 | BD Biosciences | 563033 |
| CD69 | FN50 | BV605 | Biolegend | 310937 |
